# Is the Influence of Freud Declining in Psychology and Psychiatry? A Bibliometric Analysis

**DOI:** 10.3389/fpsyg.2021.631516

**Published:** 2021-02-18

**Authors:** Andy Wai Kan Yeung

**Affiliations:** Oral and Maxillofacial Radiology, Applied Oral Sciences and Community Dental Care, Faculty of Dentistry, University of Hong Kong, Hong Kong, China

**Keywords:** citation analysis, Freud, psychoanalysis, psychiatry, psychology

## Abstract

Sigmund Freud is occasionally perceived as outdated and his work no longer relevant to academia. The citing papers (CPs) that cited Freud works were collected from Web of Science and analyzed. The 10 most common research areas of the CPs were noted, and the overall volume of the respective bodies of literature were retrieved. I computed the annual percentage of the respective bodies of literature that cited Freud. On a separate note, I computed the annual percentage of citations coming from psychology and psychiatry. Results based on 42,571 CPs found that psychology accounted for over half of the citations to Freud. The percentage of psychology papers citing Freud declined gradually from around 3% in the late 1950s to around 1% in the 2010s, in an extent of −0.02% per year over the entire survey period spanning across 65 years from 1956 till 2020 (*P* < 0.001). In psychiatry, a similar decline was observed, from around 4–4.5% in the late 1950s to just below 0.5% in the 2010s, in an extent of −0.1% per year (*P* < 0.001). However, a reverse trend was observed for psychoanalysis literature, which generally increased from 10–20% before the 1980s to 25–30% since the 2000s, in an extent of +0.2% per year (*P* < 0.001). Meanwhile, the annual percentage of CPs coming from psychology and psychiatry was decreasing by 0.4% per year (*P* < 0.001). Bibliometric data supported the notion that Freud's influence was on a decline in psychology and psychiatry fields.

## Introduction

Sigmund Freud was named as the most frequently cited psychologist of the 20th century both in the professional psychological journal literature and in introductory psychology textbooks (Haggbloom et al., 2002). Based on data from Google Scholar, it was estimated that Freud received thrice the citations of Charles Darwin, five times of Carl Jung, and six times of William James (Diener et al., 2014). He is the father of psychoanalysis, a subject of theories and therapeutic techniques that aims to treat mental disorder through the unconscious mind (Wallerstein, 1988). On the other hand, Freud and psychoanalysis are highly controversial in the modern era and have resulted in many debates and criticisms; some went as far as arguing that Freud and his theories set back the advancement of psychology and psychiatry by at least 50 years (Eysenck, 1991), and should be totally rejected by the field and not taught in universities (Simón, 2020). Psychoanalysis did suffer from an apparent decline in psychiatry in the United States in the mid-1980s, attributable to the accumulation of little empirical support (Hale Jr, 1995; Crews, 1996). Interestingly, a survey on American undergraduate education found that most of the psychoanalysis courses were not taught in the psychology department (Redmond and Shulman, 2008). In fact, 60% of courses with psychoanalytic content were taught in a humanities department, whereas 14% were in psychology and 17% were in social sciences (excluding psychology), respectively (Redmond and Shulman, 2008). Psychoanalysis was also said to be internalized by the humanities as a way to understand literature and history (Paris, 2017).

It was largely unclear if the abovementioned opinions and changes in the professional education and practice would be reflected in the academic citations. Similar studies that used citation concept analysis (CCA) have been already published to evaluate the citations received by particular books (Bornmann et al., 2020; Crothers et al., 2020). In other words, two research questions were raised:

(1) With the expanding literature in academia, did the percentage of psychology and psychiatry papers (and other disciplines) citing Freud's works decrease over the years?
(2) Among the citations made to Freud's works, did the percentage of citations from psychology and psychiatry papers decrease over the years?

This work aimed to answer these questions by exploring the bibliometric data.

## Materials and Methods

The electronic literature database Web of Science (WoS) Core Collection was accessed on 19 October 2020. A cited reference (CR) search was performed with “Freud S^*^” as the cited author. Since Freud qualified as a doctor of medicine in 1881 and passed away in 1939, I decided to limit the cited years to be 1880–1950. There were 20,509 CRs authored by “Freud S^*^.” WoS allows users to select a maximum of 1,000 CRs each time, so a series of queries were made to select all CRs. In total, there were 42,571 citing papers (CPs) that cited the CRs. These CPs were added to a Marked List in WoS, and then downloaded in full record with references in the format of plain text for analysis. WoS allows users to download per 500 papers, and thus a series of downloads were made for complete collection. Due to the limitation of the authors' institutional subscription plan, the coverage of the CPs could only date back to 1956.

In counting the frequency of countries, England, Scotland, Northern Ireland, and Wales were merged as United Kingdom. Germany and its historical derivatives, e.g., Federal Republic of Germany, West Germany, East Germany, were merged together as Germany.

To answer the first question (did the percentage of psychology and psychiatry papers citing Freud's works decrease over the years), I noted the annual frequency count of the CPs assigned to the research area of psychology (Research Areas tagged by WoS, or SU = Psychology as expressed in code), and also noted the annual publication count of WoS-indexed papers assigned to the research area of psychology. The annual percentage of psychology papers citing Freud's work could then be computed. The percentage was similarly computed for psychiatry, and the rest of the top 10 research areas.

To answer the second question (did the percentage of citations from psychology and psychiatry papers decrease over the years), I computed the annual percentage of CPs from research areas of psychology and psychiatry to the total CPs of Freud in each year.

To test for significant linear trends, linear regressions of the annual percentages were conducted in SPSS 26.0 (IBM, NY, USA). The annual percentage was inputted as the outcome variable whereas the year was inputted as the independent variable. Results were significant if *p* < 0.05.

In addition, I mapped the keywords tagged to the CPs by WoS (known as Keywords Plus) with the “Thematic Map” function of Biblioshiny using the default settings (Aria and Cuccurullo, 2017). The most frequent 200 keywords were analyzed. A strategic diagram was plotted according to Cobo et al., with four quadrants considering density and centrality: (1) upper right quadrant contains themes that are well-developed and important for the structuring of the research field with strong centrality and high density; (2) upper left quadrant contains themes that are specialized; (3) lower left quadrant contains themes with low density and centrality that are marginal and may represent emerging or disappearing themes; and (4) lower right quadrant contains themes that are important but not developed, implying transversal and basic themes (Cobo et al., 2011).

## Results

The top 10 research areas contributing to the 42,571 CPs of Freud's works are listed in Table 1. Psychology accounted for over half of the citations. Recurring major citing countries across the research areas were the United States, United Kingdom, Germany, and Canada. The top citing journal was *International Journal of Psychoanalysis*. In WoS, I found that some papers published in this journal were separately indexed under another journal name of *International Journal of Psycho-analysis* (with a hyphen). Therefore, the reported number in Table 1 was the summation of the above two, and I contacted Clarivate Analytics to rectify this.

**Table 1:** Top 10 research areas citing Freud's works.

**Research area**	**No. of citing papers (% of 42,571)**	**Top 3 authors (n)**	**Top 3 countries (n)**	**Top 3 journals (n)**
Psychology	23,942 (56.2)	Blum HP (65) Blatt SJ (51) Greenberg J (45)	US (9,239) Germany (2,394) UK (1,902)	Int J Psychoanal (2,901) J Am Psychoanal Assoc (1,925) Psyche (1,232)
Psychiatry	9.407 (22.1)	Ammon G (40) Blum HP (27) Gabbard GO (27)	US (3,704) UK (649) Germany (786)	J Am Psychoanal Assoc (1,925) Contemp Psychoanal (641) Am J Psychother (332)
Literature	2,315 (5.4)	Brooks P (8) Flieger JA (5) 13 authors (4)	US (942) UK (191) Canada (116)	Mod Lang Notes (33) James Joyce Q (32) Lit Psychol/Mosaic/New Lit Hist (30)
Social sciences other topics	1,701 (4.0)	Zwart H (12) Frosh S (10) Cotti P/Devries MFRK/Janssen DF (7)	US (600) UK (298) Canada (104)	Psychoanal Cult Soc (203) Psychoanal Hist (86) Hum Relat (79)
Arts humanities other topics	1,165 (3.8)	Sciacchitano A (9) Spitz EH (8) Zeligs DF (7)	US (612) UK (122) Canada (65)	Am Imago (594) Aut Aut (59) Semiotica (51)
Neurosciences neurology	1,446 (3.4)	Markowitsch HJ (12) Gottesmann C (9) six authors (7)	US (419) Germany (299) UK (125)	J Nerv Ment Dis (234) Nervenarzt (126) Fortschritte der Neurologie Psychiatrie (79)
Philosophy	844 (2.0)	Oliver K (7) Dilman I/Grunbaum A/Lambertino A (4)	US (245) UK (60) Italy (51)	Philos Psychiatry Psychol (37) Philos Today (22) J Conscious Stud (20)
Social work	769 (1.8)	Saari C (8) Palombo J (6) Smith M (6)	US (450) UK (69) Canada (23)	Clin Soc Work J (235) Am J Orthopsychiatry (93) Smith Coll Stud Soc (87)
Business economics	704 (1.7)	Gabriel Y (15) Devries MFRK (10) Brown AD (7)	US (301) UK (161) France (54)	Hum Relat (79) Organ Stud (27) Leadersh Q (20)
Sociology	690 (1.6)	Turner RH (4) 10 authors (3)	US (321) UK (79) Australia (23)	J Sci Study Relig (37) Am J Sociol (19) Am Sociol Rev (18)

*US, United States; UK, United Kingdom. There are multiple journals called Psyche. In here, Psyche refers to “Psyche—Zeitschrift für Psychoanalyze und ihre Anwendungen”*.

**Figure 1:**
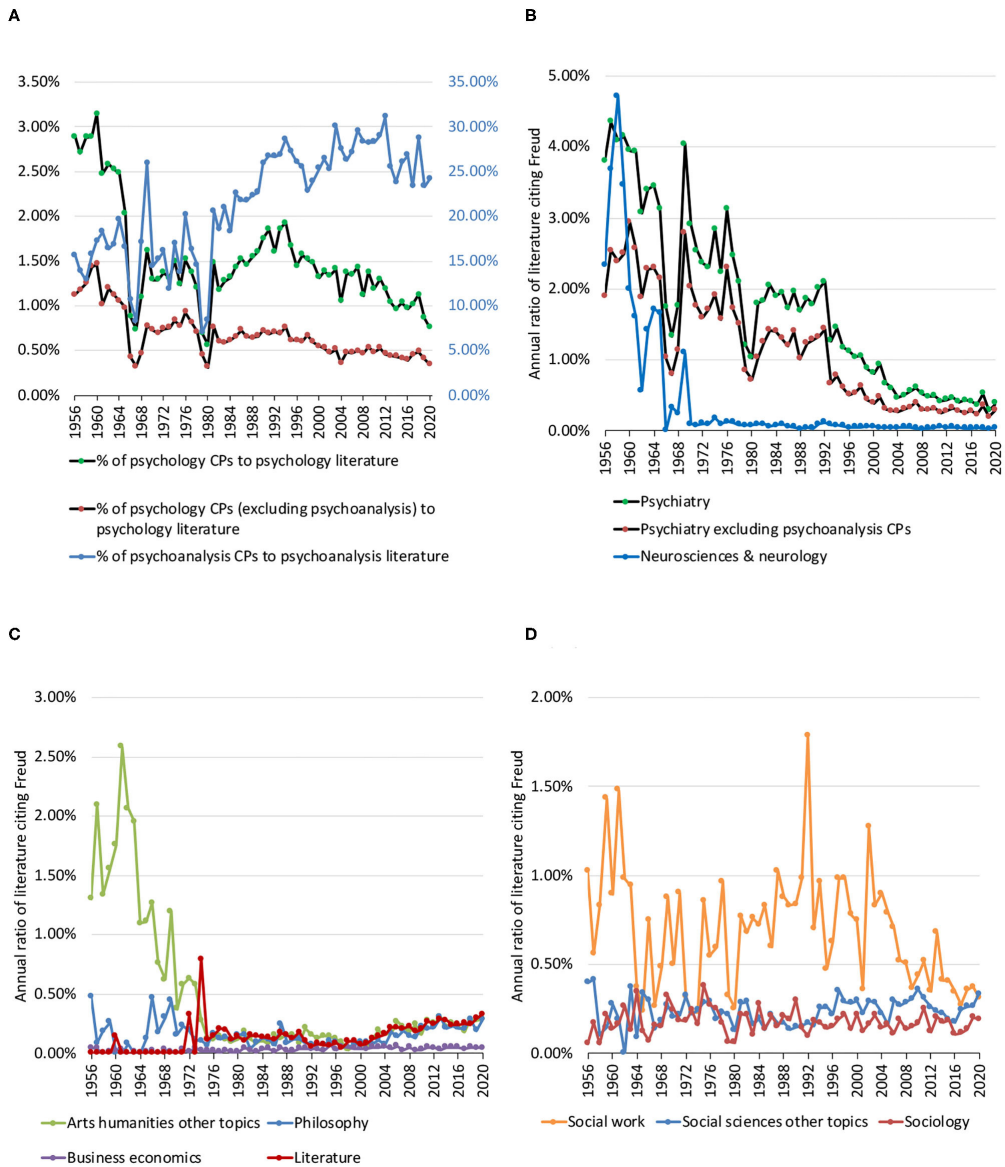
**(A)** Percentage of psychology papers citing Freud. Green dots indicate the overall percentage of psychology citing papers (CPs) to overall psychology literature. Red dots indicate the percentage, excluding CPs in psychoanalysis journals. Blue dots indicate the percentage of psychoanalysis CPs to psychoanalysis literature. **(B)** Percentage of psychiatry and neuroscience papers citing Freud. Green dots indicate the overall percentage. Red dots indicate the percentage, excluding psychiatry CPs also assigned to the category of psychoanalysis. Blue dots indicate the percentage of neurosciences and neurology. **(C)** Percentage of arts humanities other topics, business economics, literature, and philosophy papers citing Freud. **(D)** Percentage of social work, social sciences other topics, and sociology papers citing Freud.

The percentage of psychology papers citing Freud declined gradually from around 3% in the late 1950s to around 1% in the 2010s (Figure 1A, green), in an extent of −0.02% per year over the entire survey period spanning across 65 years from 1956 till 2020 (*P* < 0.001, Table 2). The percentage was halved if I excluded CPs from psychoanalysis (Figure 1A, red), in an extent of −0.01% per year (*P* < 0.001). When I focused on the psychoanalysis literature, I found that the percentage of psychoanalysis papers citing Freud generally increased from 10 to 20% before the 1980s to 25–30% since the 2000s (Figure 1A, blue), in an extent of +0.2% per year (*P* < 0.001).

**Table 2:** Trends in the percentage of literature citing Freud.

**Literature category**	**% Change per year**	***P*-value**
Psychology	−0.02	<0.001
Psychology excluding psychoanalysis CPs	−0.01	<0.001
Psychoanalysis	+0.2	<0.001
Psychiatry	−0.1	<0.001
Psychiatry excluding psychoanalysis CPs	−0.04	<0.001
Neurosciences and neurology	−0.03	<0.001
Literature	+0.004	<0.001
Arts humanities other topics	−0.02	<0.001
Philosophy	+0.0003	0.637
Business economics	+0.0006	<0.001
Social work	−0.004	0.037
Social sciences other topics	+0.0006	0.245
Sociology	−0.0003	0.505

*Linear regression was conducted for each category. CPs, citing papers. Results were significant if P < 0.05. If Bonferroni correction was applied to the results due to multiple testing (13 tests listed here), corrected results would be significant if P < 0.003. Most of the results would remain unaffected except that results for social work would become insignificant*.

In psychiatry, the decline in the percentage of papers citing Freud was similarly observed, from around 4–4.5% in the late 1950s to just below 0.5% in the 2010s (Figure 1B, green), in an extent of −0.1% per year (*P* < 0.001). This decline percentage was the largest among the top 10 research areas. Psychiatry CPs assigned to psychoanalysis were not as many as in psychology, and thus excluding them did not lead to such a huge effect as in psychology (Figure 1B, red), in an extent of −0.04% per year (*P* < 0.001).

In neurosciences and neurology, the percentage of papers citing Freud was on a decline with −0.03% per year (*P* < 0.001). The decline was particularly prominent from the mid-1950s to the end of the 1970s (Figure 1B, blue), and since then the annual percentage was always <0.15%.

Outside behavioral sciences, the trends in the percentages of papers citing Freud varied. In arts humanities other topics, the percentage declined from the mid-1950s to the end of 1970s in a similar fashion to neurosciences and neurology and had an overall decline trend (*P* < 0.001), whereas literature and business economics had an overall increasing trend (both *P* < 0.001). These trends had a smaller magnitude than those for behavioral sciences (Table 2). Overall trend for philosophy was not significant (*P* = 0.637), though from Figure 1C it could be observed that it had a rising curve since 2000, together with literature and arts humanities other topics.

In social sciences, social work had an overall declining trend of citing Freud (−0.004% per year, *P* = 0.037), whereas social sciences other topics and sociology had non-significant trends (Table 2; Figure 1D).

Therefore, the answer to the first research question (did the percentage of psychology and psychiatry papers citing Freud's works decrease over the years) was yes. For other disciplines, the trends varied but their magnitudes of change were much smaller than for psychology and psychiatry.

**Figure 2:**
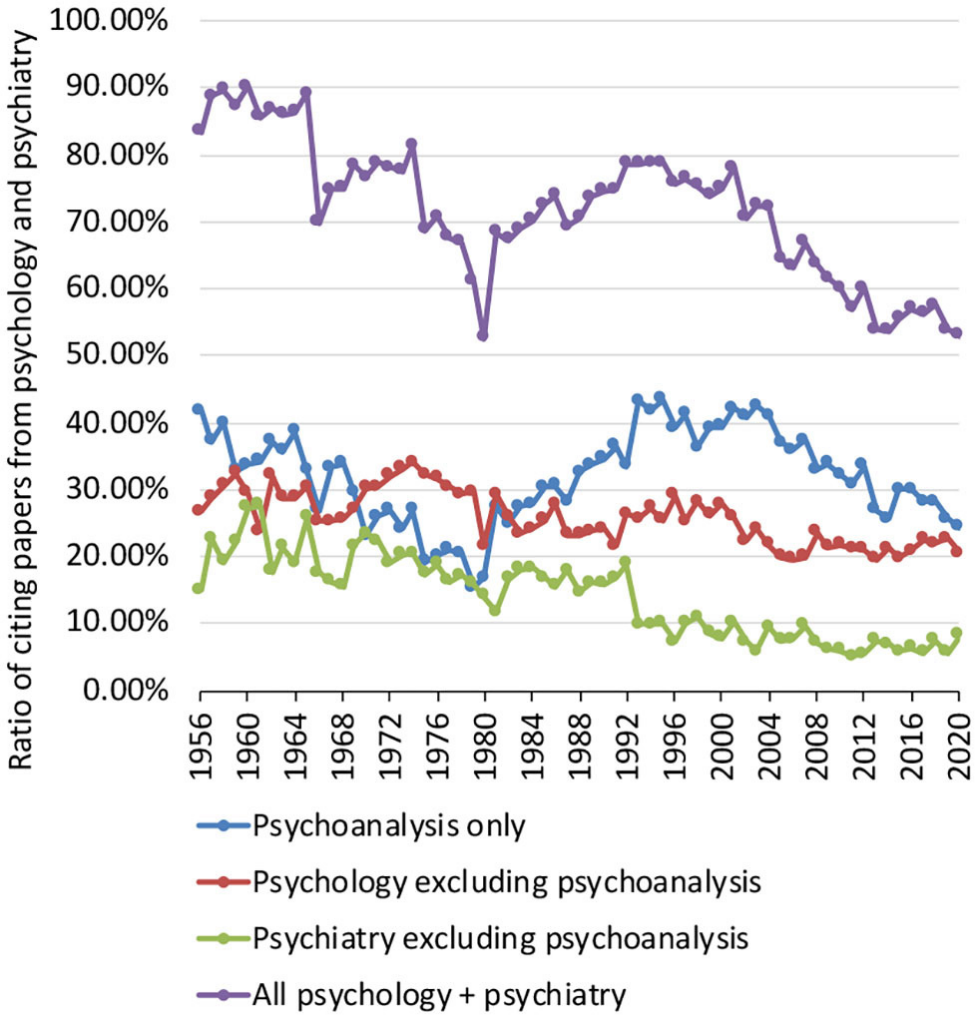
Annual percentage of citing papers coming from psychology and psychiatry. Four percentages were computed: overall percentage considering psychology and psychiatry together (purple), psychoanalysis only (blue), psychology (red), and psychiatry (green) without psychoanalysis respectively.

Then, the annual percentage of CPs coming from psychology and psychiatry was computed (Figure 2). In overall, this percentage decreased by 0.4% per year over the 65-year survey period (*P* < 0.001). Probing into the data, the percentage of CPs coming from psychology (excluding psychoanalysis) decreased by 0.2% per year (*P* < 0.001); the percentage coming from psychiatry (excluding psychoanalysis) decreased by 0.3% per year. The percentage did not show a significant trend for CPs coming from psychoanalysis (0.03 per year, *P* = 0.506).

The answer to the second research question (did the percentage of citations from psychology and psychiatry papers decrease over the years), therefore, was also yes.

In addition, I plotted a graph of citations gained by the CPs against publication year (Supplementary Figure 1A). Results showed that citations to CPs of Freud showed an increasing trend since 1968, and after 1995 the decline was as expected. When the publication and citation counts of the CPs were examined further, I found that the annual publication count of CPs actually increased steadily, whereas the citations per publication was the opposite (Supplementary Figure 1B).

Thematic map (Supplementary Figure 2) showed that the motor themes (well-developed and important for the structuring of the research field with strong centrality and high density) included two clusters of keywords: (1) depression theme, with depression (*n* = 470), children (384), therapy (269), trauma (243), stress (232), disorder (225), disorders (221); and (2) behavior theme, with behavior (526), model (395), personality (386), and women (250). Themes that were emerging or disappearing (low density and low centrality) included two clusters: (3) self theme, with self (549), memory (334), psychology (283), and brain (230); and (4) psychoanalysis theme, with psychoanalysis (621), psychotherapy (579), transference (392), attachment (306), countertransference (283), and experience (279).

## Discussion

By analyzing the distribution of 42,571 CPs spanning from year 1965 to 2020, I confirmed the notion that Freud's influence has been on a decline in the academia of psychology and psychiatry in terms of declining citation percentages. However, readers should interpret the findings cautiously, as the small declining trend might not be solely attributed to Freud's relevance, but also other effects such as “obliteration by incorporation” (Merton, 1965). Reduced citation due to obliteration by incorporation was observed in other topics such as Thomas Kuhn's model of paradigm shift (Marx and Bornmann, 2010) and John Nash's famous Nash equilibrium (Mccain, 2011).

Psychoanalysis itself certainly is still a vibrant research field, with multiple journals dedicated to this subject, such as *American Imago* (co-founded by Freud), *Journal of the American Psychoanalytic Association, International Journal of Psychoanalysis, Psyche—Zeitschrift für Psychoanalyze und ihre Anwedungen, Psychoanalytic Quarterly*, and *Contemporary Psychoanalysis*, to name a few. Freud is the father of psychoanalysis (Blum, 1990), and during the survey period one-fourth to nearly one half of his annual citations came from psychoanalysis papers. From another perspective, in each year 10–30% of the entire psychoanalysis literature cited Freud. Therefore, the argument (or prediction), made during the second half of the 20th century, that the psychoanalysis was “dead” in terms of a theoretical system and a treatment modality seemed not to be materialized (Conn, 1973). The propositions of constraints on connectionist models for cognitive science brought about by unconscious affective and motivational dynamics might have regained relevance in psychological science (Westen, 1998, 1999).

There was an opinion that modern neuroscience probing into consciousness and mind with the use of neuroimaging modalities, such as functional magnetic resonance imaging, might have revived the concepts constructed by Freud (Berlin and Koch, 2009; Owen, 2017). In particular, there is a notion that transiting from unconsciousness to consciousness is an increase in complexity and involves affective dynamics that might be characterized by free energy principle and integrated information processing (Carhart-Harris and Friston, 2010; Friston, 2010; Tononi et al., 2016; Solms, 2019). It seemed that Freud's interest in the unconsciousness theme could be further developed by neurosciences. However, the results from the current study failed to substantiate the claim. The annual count of CPs (both absolute value and relative percentage) failed to reflect an increase in citing Freud by neurosciences papers in the 21st century. Perhaps the influence existed in the forms of scientific blogs and social media activities but not as journal citations. This situation could remotely resemble living scientists with many followers for their social media profiles but a relatively low h-index, or like a celebrity (Hall, 2014). At least the current data did not suggest a rise in popularity of Freud's ideas in the neuroscience field.

The resemblance of Freud as a celebrity could be further argued by the fact that people not only study his research work but also probes into every aspect of his life. For instance, his written letters were extensively appreciated in terms of his word choice, handwriting, tone, and content (Grotjahn, 1967). His essay was similarly analyzed in terms of how his concepts were explained, how he clarified issues with self-arguments, and so on (Cixous, 1976). Freud is also being related to remotely. For example, in an analysis of the fiction The Hunger Games, Freud was cited for his concepts on narcissism and Oedipus complex (Manter and Francis, 2017). A television series aired in 2020 had a plot that re-imagined how a young Freud solved mysteries in the city of Vienna (https://www.imdb.com/title/tt8667956/). These examples were accompanied with an increasing trend in citing Freud in arts humanities and literature publications especially since the 2000s. It implied that while Freud might have lost his niche in psychology and psychiatry fields, he has gained it in arts.

The current study has several limitations. The WoS Core Collection database might not index all CRs and CPs. When I searched for the annual publication count of various research areas, I noticed that several areas had a sudden drastic increase at particular years, suggesting a wider coverage/indexing since a certain year. For instance, the annual publication count of the research area literature drastically increased from 126 in 1974 to 25,635 in 1975; arts humanities other topics drastically increased from 1,790 in 1974 to 8,758 in 1975; and neurosciences and neurology drastically increased from 817 in 1969 to 5,385 in 1970. The sudden increase in the publication count might “dilute” the percentage of CPs of Freud if WoS did not also increase the indexing of CPs accordingly. This might partially explain the abrupt change in the curve form for the latter two research areas. Scopus may have a better coverage of social sciences literature (Norris and Oppenheim, 2007). Notwithstanding, it was reported that both databases provided datasets that led to comparable bibliometric results from cross-disciplinary comparisons including social sciences (Harzing and Alakangas, 2016). Another limitation was that the search for “Freud S^*^” could capture authors other than Sigmund Freud. The initial thought was to search for the exact name of Sigmund Freud, but many old publications were indexed with the author's first name initialized. To strike a better balance, the finalized search involved “Freud S^*^” but limited to references published during 1880–1950. Finally, I did not investigate the contextual basis of the CPs, i.e., whether their citations were supportive, neutral, or contradicting. The contextual basis of the citations could be further evaluated in future studies by CCA by utilizing the data from databases that store the semantic content of the citations, such as Microsoft Academic.

## Conclusion

The study on 42,571 papers found a decreasing trend in citing Freud by psychology, psychiatry, and neuroscience papers, but an increasing trend in arts humanities, literature, and business economics especially since the 2000s. The decline in psychiatry had the largest magnitude among the trends, in an extent of −0.1% per year over a 65-year time span. The annual percentage of citations coming from psychology and psychiatry was decreasing at a percentage of 0.4% per year. Outside the study of psychoanalysis, Freud's influence in psychology and psychiatry has been reducing.

## Data Availability Statement

The data analyzed in this study is subject to the following licenses/restrictions: Data can be provided on considerable request, since it was extracted from subscription-based database. Requests to access these datasets should be directed to Andy Yeung, ndyeung@hku.hk.

## Author Contributions

The author is responsible for all parts of the work.

## Conflict of Interest

The author declares that the research was conducted in the absence of any commercial or financial relationships that could be constructed as a potential conflict of interest.
