# Supplementary material for: Is the Influence of Freud Declining in Psychology and Psychiatry? A Bibliometric Analysis
Source: Front Psychol. 2021 Feb 18;12:631516. doi: 10.3389/fpsyg.2021.631516 (PMC7930904; doi:10.3389/fpsyg.2021.631516)

Supplementary Figure 1. (A) Citation count of the citing papers (CPs) of Freud against publication year. (B) Annual publication count and citations per publication of CPs of Freud. PY, publication year. TC, total citation. c/p, citation/publication. Source: Professor Loet Leydesdorff, University of Amsterdam, Amsterdam School of Communication Research (ASCoR), private communication.

(A)

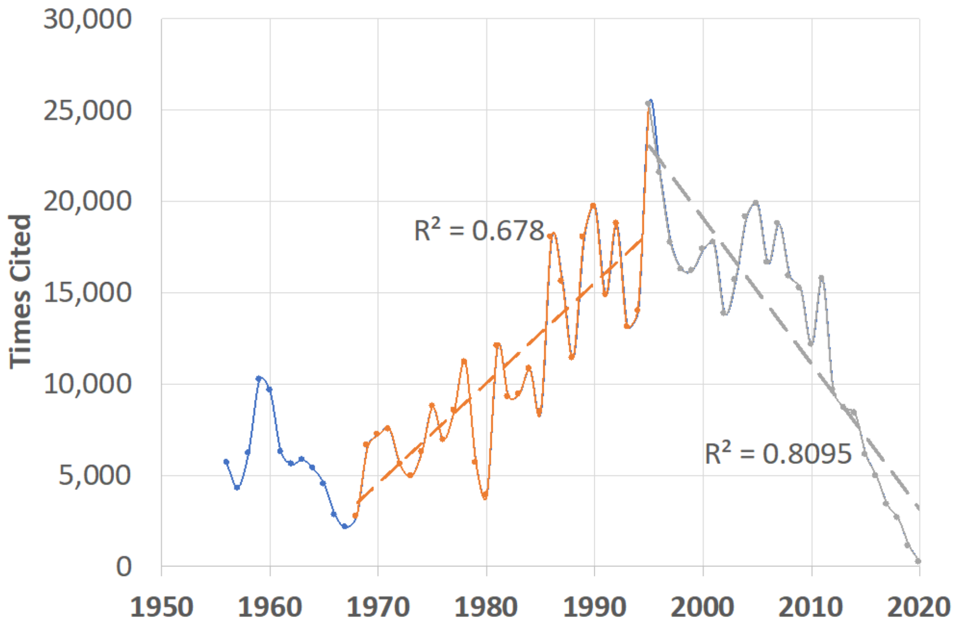

(B)

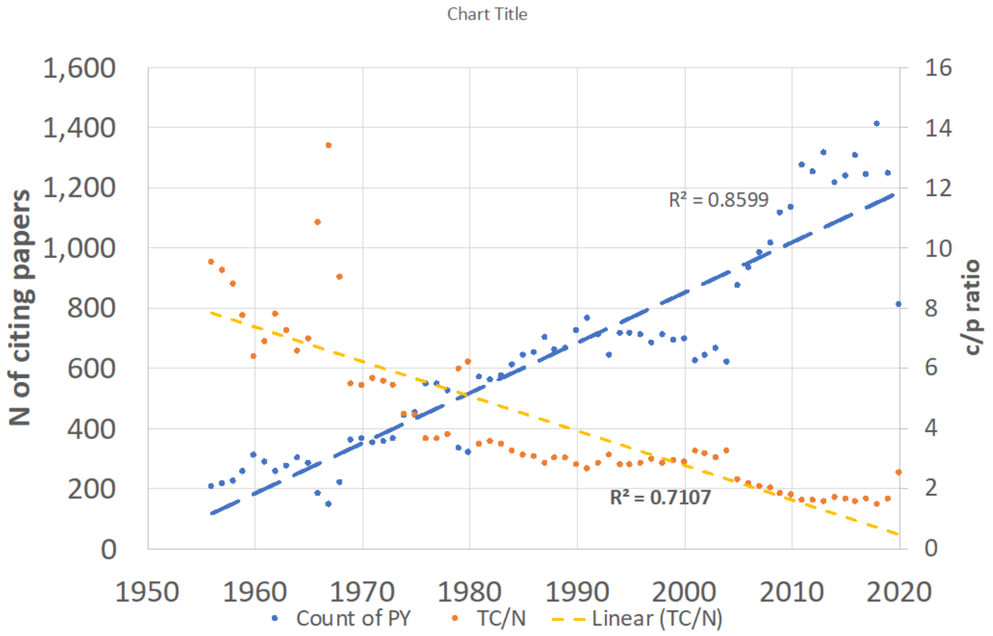

Supplementary Figure 2. Thematic map of citing papers of Freud.

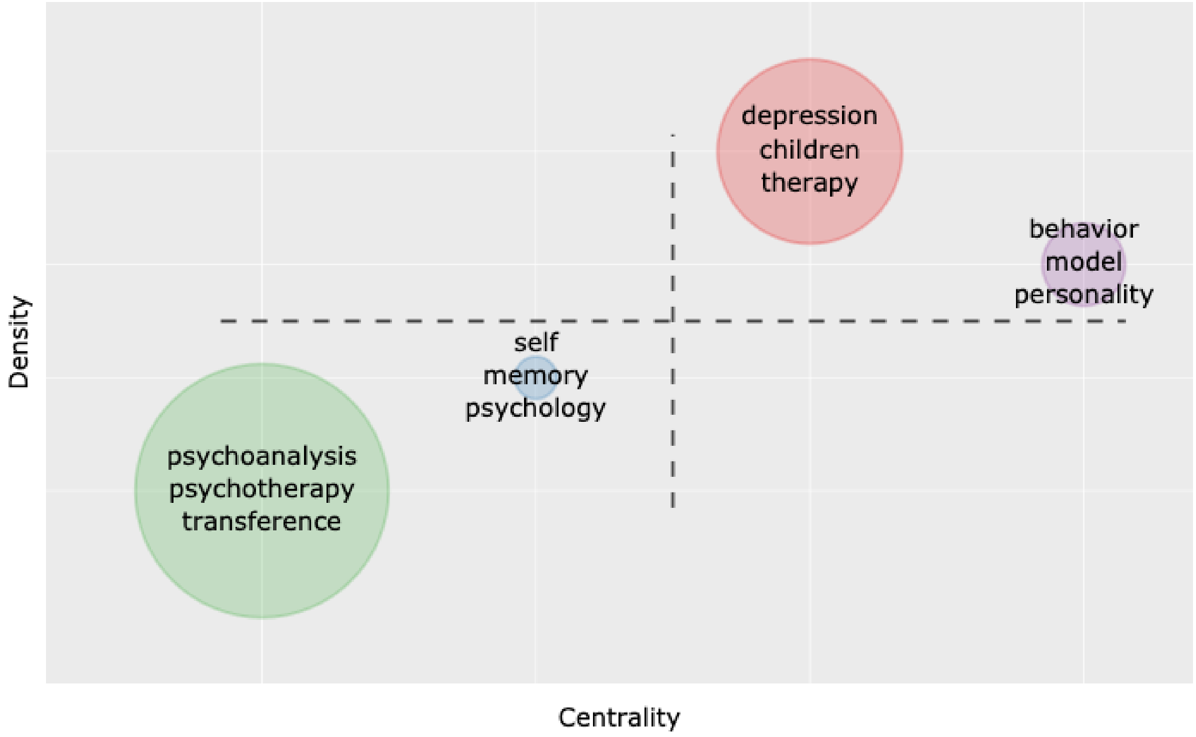

Supplement: Supplementary file 1 [file Image_1.pdf]
